# Unmasking hidden risks: The surprising link between PDE5 inhibitors and seizure susceptibility

**DOI:** 10.1371/journal.pone.0294754

**Published:** 2023-11-30

**Authors:** Alex Luiz Menezes da Silva, Chirlene Pinheiro Nascimento, Julianne Elba Cunha Azevedo, Luana Rodrigues Vieira, Akira Otake Hamoy, Allan Carlos da Silva Tiago, João Cleiton Martins Rodrigues, Daniella Bastos de Araujo, Dielly Catrina Favacho Lopes, Vanessa Jóia de Mello, Moisés Hamoy

**Affiliations:** 1 Laboratory of Pharmacology and Toxicology of Natural Products, Institute of Biological Sciences, Federal University of Pará, UFPA, Belém, Pará, Brazil; 2 Laboratory of Experimental Neuropathology, Institute of Biological Sciences, Federal University of Pará, UFPA, Belém, Pará, Brazil; Massachusetts General Hospital, UNITED STATES

## Abstract

**Background:**

Phosphodiesterase 5 inhibitors (PDE5i) are the first line treatment for erectile dysfunction; however, several articles and case reports have shown central nervous system effects, that can cause seizures in susceptible patients. This study aims to describe the changes caused by the use of Sildenafil and Tadalafil through the analysis of abnormalities expressed in the electrocorticogram (ECoG) of rats and evaluate the seizure threshold response and treatment of seizures with anticonvulsants.

**Materials and methods:**

The study used 108 rats (Wistar). Before surgery for electrode placement in dura mater, the animals were randomly separated into 3 experiments for electrocorticogram analysis. Experiment 1: ECoG response to using PD5i (Sildenafil 20mg/kg and Tadalafil 2.6mg/kg p.o.). Experiment 2: ECoG response to the use of PD5i in association with Pentylenetetrazole (PTZ—30 mg/kg i.p.), a convulsive model. Experiment 3: ECoG response to anticonvulsant treatment (Phenytoin, Phenobarbital and Diazepam) of seizures induced by association IPDE5 + PTZ. All recordings were made thirty minutes after administration of the medication and analyzed for ten minutes, only once. We considered statistical significance level of *p<0.05, **p<0.01 and ***p < 0.001.

**Results:**

After administration of Sildenafil and Tadalafil, there were increases in the power of recordings in the frequency bands in oscillations in alpha (p = 0.0920) and beta (p = 0.602) when compared to the control group (p<0.001). After the use of Sildenafil and Tadalafil associated with PTZ, greater potency was observed in the recordings during seizures (p<0.001), however, the Sildenafil group showed greater potency when compared to Tadalafil (p<0.05). Phenobarbital and Diazepam showed a better response in controlling discharges triggered by the association between proconvulsant drugs.

**Conclusions:**

PDE5i altered the ECoG recordings in the rats’ motor cortexes, demonstrating cerebral asynchrony and potentiating the action of PTZ. These findings demonstrate that PDE5i can lower the seizure threshold.

## Introduction

Phosphodiesterase 5 (PDE5) isoenzymes have been identified in a wide variety of tissues, e.g., the smooth muscle cells of the corpus cavernosum, vascular and visceral smooth muscle, skeletal muscle, platelets, kidney, lung, spinal cord, cerebellum, pancreas, prostate, urethra, and bladder [1–3].

Current guidelines for the treatment of erectile dysfunction recommend PDE5 inhibitors as the first-line therapy for most men with erectile dysfunction who do not have a specific contraindication to their use [4]. They state that PDE5 inhibitors are effective, safe, and well-tolerated therapies and that there are no significant differences in efficacy, safety, and tolerability between the approved drugs [4].

Sildenafil was the first (in 1998) compound introduced clinically, followed by Vardenafil and Tadalafil (in 2003) and Avanafil (in 2013) [5]. Studies in vitro have shown that Sildenafil’s inhibition of PDE5 is 10-fold greater than that of PDE6, 100-fold greater than that of PDE1 and 1000-fold greater than that of PDE2, PDE3, and PDE4 [6].

PDE5 inhibitors can cause side effects in the central nervous system by crossing the blood-brain barrier [7]. Sildenafil induces transiently increased glutamate levels in the brainstem, which suggests transiently increased excitability of the brainstem neurons [8]. Several articles and case reports have shown some relationship between increased neuronal excitability and even proconvulsant properties associated with using PDE5 inhibitors [9–19].

De Carvalho et al. [20] noted that the seizure threshold was lowered by Sildenafil for the Pilocarpine seizure model, involving cholinergic pathways in the central nervous system.

In addition, articles point to the possibility that pro-inflammatory factors are involved in the pathophysiology of seizures [21]. Thus, Nitric oxide (NO) stands out, which can intensify oxidative stress through the overproduction of reactive oxygen species [22, 23], resulting in cell damage. Considering that PDE5 inhibitors are able to cross the blood-brain barrier [7], their NO potentiating action, increasing the GMPc, could result in convulsive effects.

According to Okuyucu et al. [24], PDE5 inhibitors can produce electroencephalogram (EEG) abnormalities. This study aims to describe the changes caused by the use of Sildenafil and Tadalafil through the analysis of abnormalities expressed in the Electrocorticogram (ECoG) and to evaluate the possible change in the epileptic threshold after the test with Pentylenetetrazole.

## Materials and methods

### Animals

This study used 108 male Wistar rats (Rattus norvegicus), aged between 100 and 120 days and weighing between 200 to 220g. The rats were kept in cages, 3 per cage, measuring 60cm x 40cm x 20cm. The environment was enriched with wood shavings as bedding material, the temperature was maintained at 22 ± 2°C, with a light-dark period of 12 hours, water and feed offered at will; Purina® brand commercial feed with 20% protein.

The experience with recording ECoG rodents from the Laboratory of Pharmacology and Toxicology of Natural Products facilitated the choice of Wistar rats for the experiments [25]. Furthermore, the physiology and cerebral vasculature of rodents are similar to that of humans, so they were selected as a study model for ischemic stroke [25]. Finally, this choice and sample number were necessary to prove the effects and allow other scientists to reproduce this experiment.

All procedures followed the current Brazilian legislation related to ethics in animal experimentation. This project was previously approved by the Animal Use Ethics Committee (CEUA / UFPA). The project’s approval number is 5735240419.

### Surgery for electrode placement

For electrode implantation, the animals were anesthetized with an association of Xylazine Hydrochloride (5mg/kg) and Ketamine Hydrochloride (50mg/kg) through intraperitoneal injection, with an initial infiltration of lidocaine (2%) at the surgical incision site. The electrodes were implanted at the Bregma -0.96mm stereotaxic coordinate [26] and ± 1mm lateral to the dura mater region just above the motor cortex [27–30]. The electrode was in contact with the surface of the dura mater. During the first two days after the implant surgery, the animals received Ketoprofen 1mg/kg i.p., and on the fifth post-surgical day, the experiment was carried out.

During anesthesia and post-surgery, the animals heart rate and temperature were monitored, with analyses done every 6 hours, for 3 minutes. The conditions of asepsis in the handling of the animals were guaranteed throughout the entire experiment.

### Experimental design

The animals were randomly separated into groups for each experiment. Rodents remained in individualized cages from electrode implantation to ECoG recordings, with the registrations being made between 8:00 and 10:00 a.m. All recordings were made thirty minutes after the administration of the medications and analyzed for a period of ten minutes, only once.

Three experiments were carried out. For each one, the following dosages were adopted: control groups received orally 0.9% saline solution; Sildenafil-treated groups received 20 mg/kg p.o. [31]; tadalafil-treated groups received 2.6 mg/kg p.o. [32]; Pentylenetetrazole (PTZ) -treated groups received 30 mg/kg i.p. [33].

#### Experiment -1

Composed of 3 analyzed groups: a) the Control group (n = 9); b) the Sildenafil group (n = 9) and c) the Tadalafil group (n = 9).

#### Experiment -2

Composed of 4 analyzed groups: a) Control group (n = 9); b) PTZ group (n = 9); c) Tadalafil and PTZ group (n = 9); and d) Sildenafil and PTZ group (n = 9). The groups were treated with Tadalafil and Sildenafil thirty minutes before the application of PTZ to assess seizure activity through ECoG in the rats.

#### Experiment -3

The groups that received Tadalafil and Sildenafil associated with PTZ were treated with anticonvulsant drugs: Phenytoin (10mg/kg i.p.), Phenobarbital (10mg/kg i.p.) or Diazepam (10mg/kg i.p.). Therefore, the groups formed were compared with each other and the control groups who received orally 0.9% saline solution.

In experiment 3, nine groups were analyzed: a) Control group (n = 9); b) Tadalafil and PTZ group (n = 9); c) Tadalafil, PTZ and Phenytoin group (n = 9); d) Tadalafil, PTZ and Phenobarbital group (n = 9); e) Tadalafil, PTZ and Diazepam group (n = 9); f) Sildenafil and PTZ group (n = 9); g) Sildenafil, PTZ and Phenytoin group (n = 9); h) Sildenafil, PTZ and Phenobarbital group (n = 9); i) Sildenafil, PTZ and Diazepam group (n = 9).

Aiming to reduce the number of animals used in the experiment, in line with the CEUA / UFPA guidelines, data obtained from equivalent groups were reused between experiments: Control group (Experiments 1, 2 and 3); Tadalafil and PTZ group (Experiments 2 and 3); and Sildenafil and PTZ group (Experiments 2 and 3).

After the registration period, the animals were euthanised by a professional veterinarian and given high doses of ketamine (300 mg/kg) and xylazine (20 mg/kg), and Diazepam (10 mg/kg) (i.p.) to avoid suffering. This follows institutional requirements for the euthanasia of these animals.

### Chemicals and reagents

The anaesthetic Ketamine was purchased from König (Santana de Parnaíba SP Brazil) and Xylazine from Vallée (Montes Claros MG Brazil), while the local anesthetic Lidocaine was obtained from Hipolabor (Sabará MG Brazil). The anticonvulsant compound Phenobarbital was purchased from Aventis-Pharma (Ribeirão Preto, SP, Brazil), and Diazepam and Phenytoin from União Química (Embu-Guaçu, SP, Brazil). The PTZ was obtained from Sigma Chemical Co. (St. Louis, MO, United States), and Sildenafil citrate 50 mg Viagra® from Pfizer (Guarulhos São Paulo–Brazil) and Tadalafil 20 mg Cialis® from Lilly (São Paulo SP- Brazil).

### Electroencephalographic records

Electrodes were connected to a digital data-acquisition system composed of a high impedance-amplifier (Grass Technologies, P511), an oscilloscope (Protek, 6510), and a board for data acquisition and digitalization (National Instruments, Austin, TX). Data was continuously sampled at 1 kHz at a low pass of 3 kHz and a high pass of 0.3 Hz. The recordings followed a standard protocol: the animals were meticulously immobilized and given a 10-minute acclimation period before the start of the recordings, all to prevent any potential interference with the recording process [27].

### Data analyses

Analysis performed offline: a double-blind controlled design through a tool built using Python programming language (version 2.7). “Numpy” and “Scipy” libraries were used for mathematical processing and the “matplolib” library was used to obtain graphs and plots. A graphic interface was developed using the PyQt4 library. Spectrograms were calculated using a Hamming window with 256 points (256/1000 s). For power spectral density (PSD), each frame was generated with an overlap of 128 points per window. For each frame, the PSD was calculated by Welch’s average periodogram method. Frequency histograms were obtained by calculating the PSD of the signal using the Hamming window with 256 points without overlap, yielding a resolution of 1Hz per bin. Each wave displayed in PSD is an average from a set of experiments. PSDs were calculated in each group, and the means are shown by individual bins [27].

### Statistical analysis

Kolmogorov-Smirnov and Levene tests were used to verify data normality and variance homogeneity, respectively. For graphics presentation and statistics calculation, prism® software was used. Comparisons of mean power values were performed using a one-way analysis of variance (ANOVA) followed by Tukey’s post hoc test, applied to aim for a statistical significance level of *p<0.05, **p<0.01 and ***p < 0.001. The data and statistical analysis complied with the recommendations on experimental design and analysis in pharmacology [34].

For the analysis of brain wave oscillations, the following frequency values were considered: delta (1.0–4.0Hz), theta (4.0–8.0Hz), alpha (8.0–12.0Hz), beta (12.0–28.0Hz) and gamma (28.0–40.0Hz).

## Results

**Fig 1:**
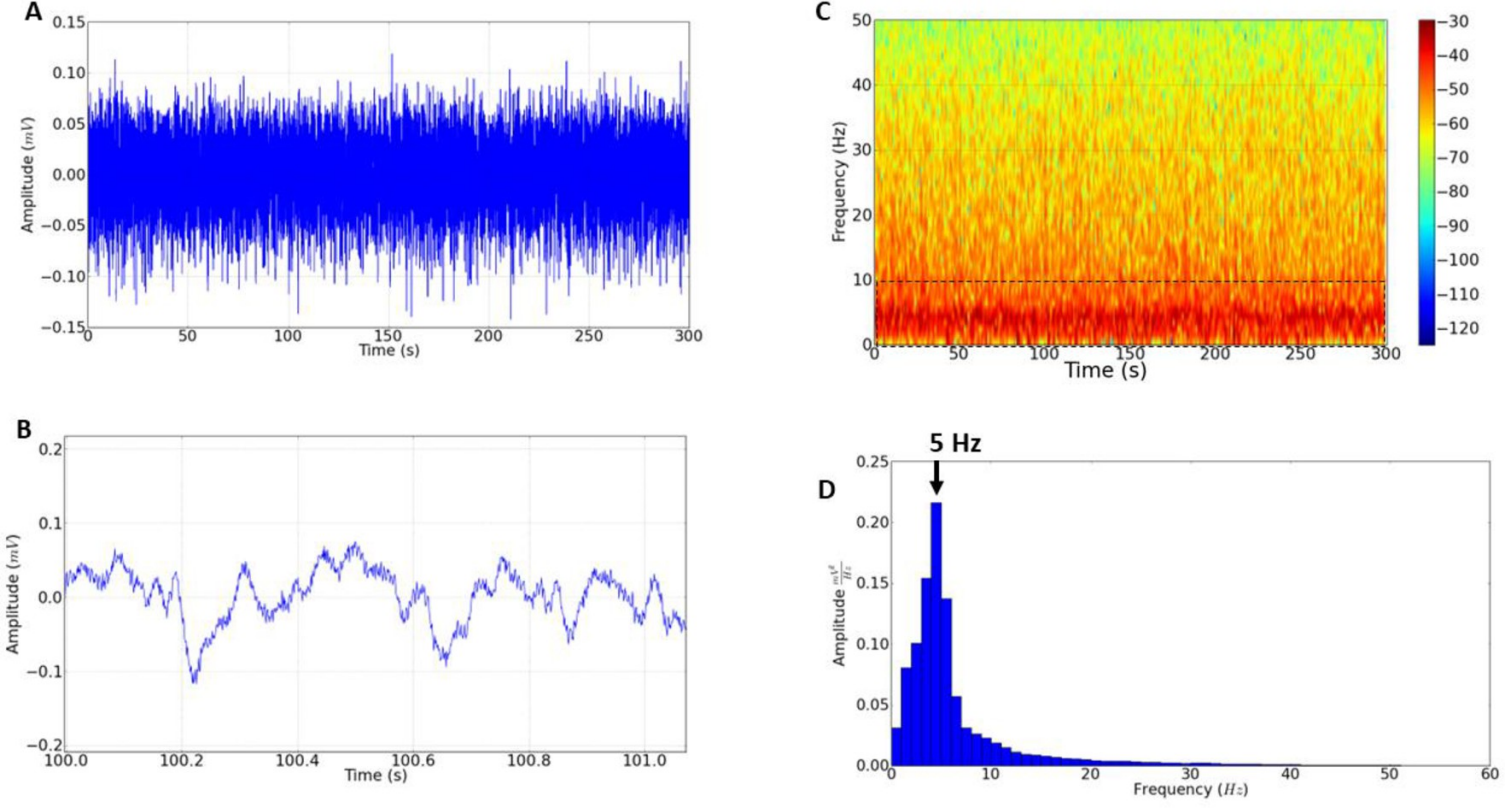
Brain activity of the control group. (A) Electrocorticographic record of the animal in basal state lasting 300 seconds (s); (B) Amplification of the electrocorticographic record in a time of 1 second (to demonstrate the control tracing pattern); (C) Spectrogram demonstrates the concentration of the spectral energy distribution in the control (indicated by dashed line); (D) Histogram shows greater energy intensity at the frequency of 5 Hz (Indicated by the arrow).

The record of the control group showed an amplitude ranging from 0.05 to 0.1mV (**Fig 1A and 1B**), and the spectrogram demonstrated higher energy intensity on frequencies between 1.0 to 10.0 Hz (**Fig 1C**). The histogram shows greater amplitude at 5.0 Hz, corroborating the energy distribution observed in the spectrogram (**Fig 1D**).

**Fig 2:**
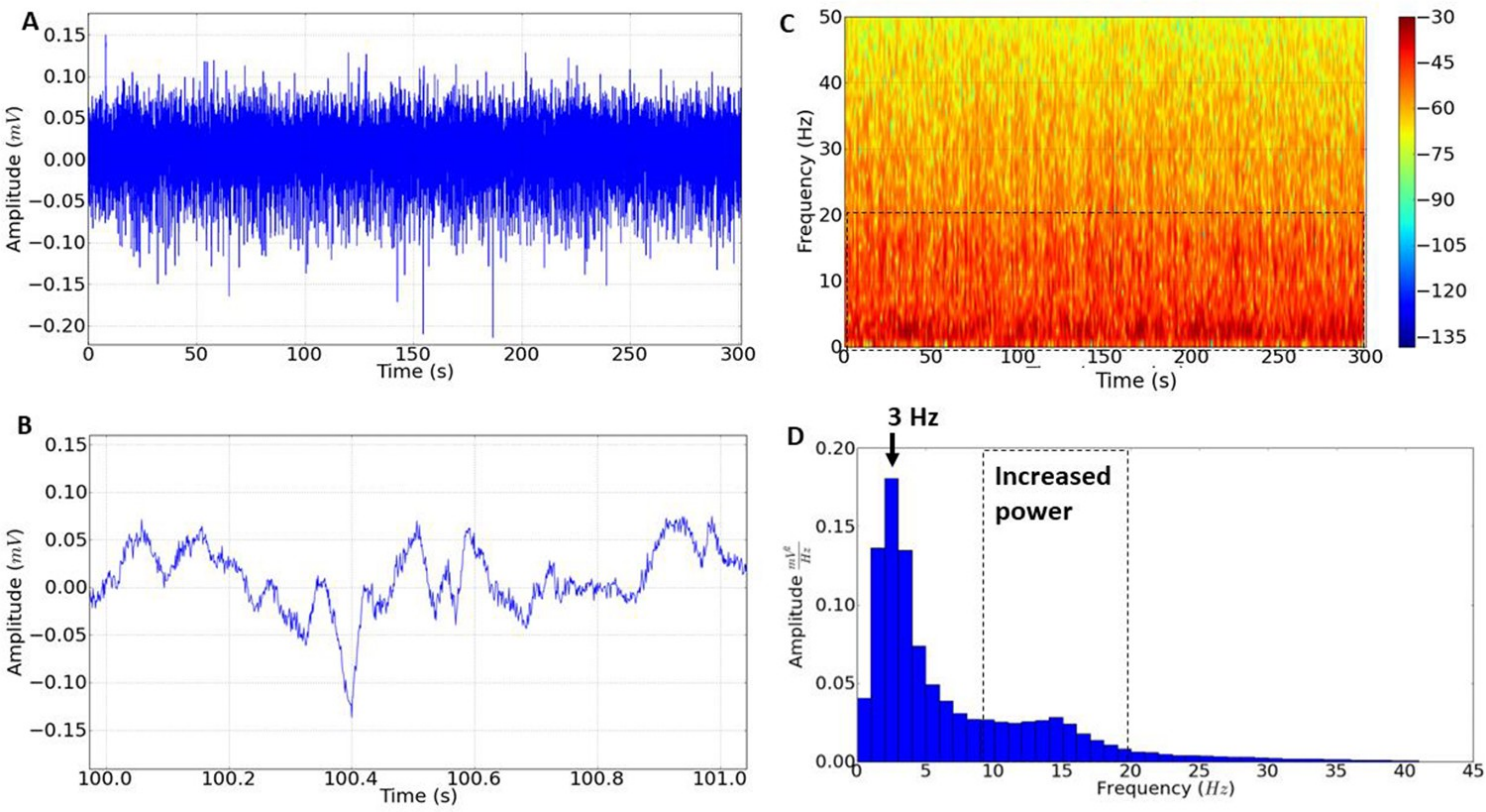
Characteristic of the electrocorticographic record after the administration of Sildenafil. (A) Electrocorticographic recording lasting 300s (demonstrating amplitude in mV); (B) Amplification of the electrocorticographic record (1.0 s) demonstrating the characteristic of frequencies and amplitude; (C) Spectrogram demonstrating energy distribution with increased cerebral asynchrony at frequencies up to 20 Hz (dashed line). (D) Amplitude distribution histogram. The predominant frequency is 3 Hz (indicated by the arrow) which shows an increase in power in the frequencies up to 20 Hz (indicated by the dashed line).

After administration of Sildenafil, the electrocorticographic record showed an amplitude variation of 0.05 to 0.1 mV, similar to that observed in the control group record (**Fig 2A and 2B**). The difference between the Sildenafil group and the control group is related to the energy distribution, which is maintained between 1.0 to 20.0 Hz, which can be observed in the spectral energy distribution (**Fig 2C**). The histogram of amplitude distribution in the frequencies individually until 40 Hz, the greatest amplitude is in the frequency of 3.0 Hz. The increase in amplitude in the frequencies between 10.0 to 20.0 Hz can also be observed after the use of Sildenafil (**Fig 2D**).

**Fig 3:**
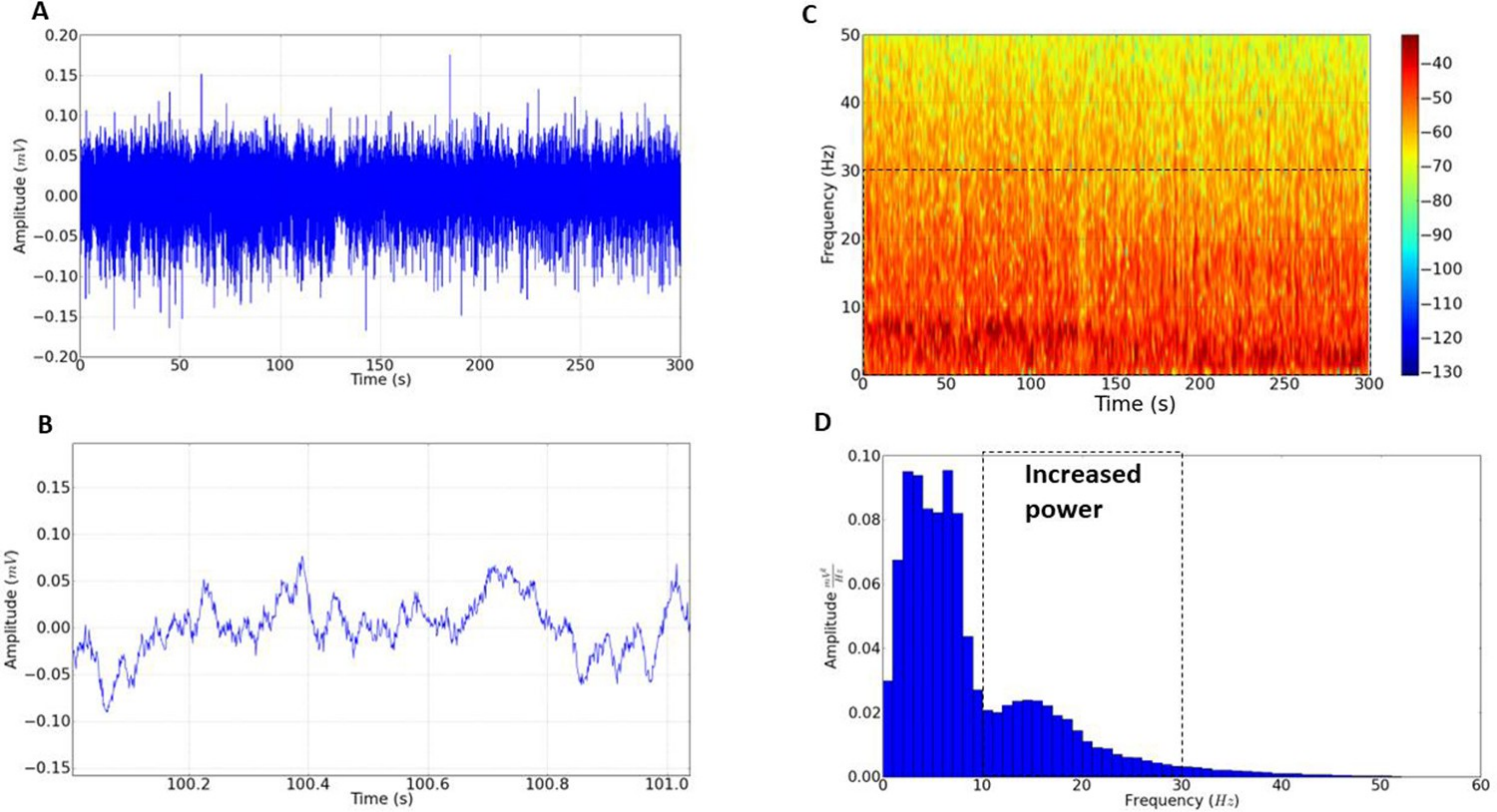
Characteristic of the electrocorticographic record after administration of Tadalafil. (A) Electrocorticographic recording lasting 300s, tracing with amplitude in mV; (B) Amplification of the electrocorticographic record (1.0 s) demonstrating the characteristic of tracing; (C) Spectrogram demonstrating energy distribution with increased cerebral asynchrony at frequencies up to 30 Hz (dashed line). (D) The amplitude distribution histogram showed a loss of predominant frequency, with increased power given in frequencies up to 30 Hz (indicated by the dashed line).

After Tadalafil application, a change in power distribution was observed at frequencies up to 30 Hz (indicated by the dashed line) (**Fig 3C**). The group treated with Tadalafil observed a greater cerebral asynchrony captured by the electrode; however, it showed amplitudes with a range similar to the control group from 0.05 to 0.1 mV (**Fig 3A and 3B**). The spectrogram showed changes in spectral powers at frequencies between 1.0 to 30.0 Hz, which caused changes in brain oscillations (**Fig 3C**). The histogram showed that the dominant amplitudes are between 2.0 to 8.0 Hz (**Fig 3D**), and there was an increase in cerebral asynchrony in the frequency bands in alpha (8–12 Hz) and beta (12–28 Hz).

Thus, the PDE inhibitors showed an increase in power in the alpha (8–12 Hz) and beta (12–28 Hz) frequency bands. However, the spectrogram demonstrates greater spectral intensity for Tadalafil (**Figs 2 and 3**).

**Fig 4:**
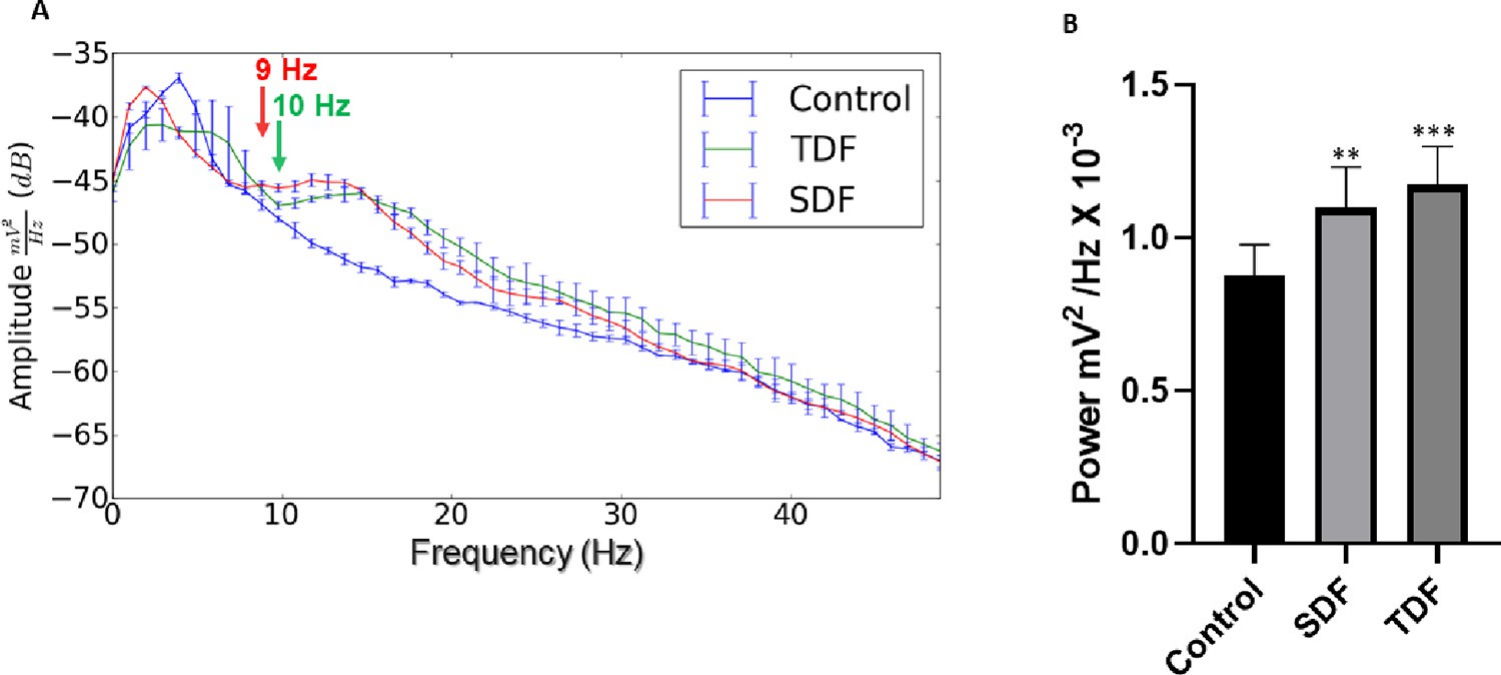
Power spectral distribution relating to the control of, Sildenafil (SDF), and Tadalafil (TDF) records. (A) Spectral distribution of power means (SD) at frequencies up to 50 Hz of the control (blue line), SDF (red line), and (TDF) (green line). The red arrow indicates the start of increased cerebral asynchrony for SDF, and the green arrow indicates the beginning of the increase in TDF cerebral asynchrony. (B) Linear power distribution at frequencies up to 50 Hz. * indicates statistical difference for the control group. (ANOVA and Tukey’s post-test) (*) p <0.01, (***) p<0.001. (n = 9).

The Fourier analysis showed a greater increase in power in a synchronized way for Sildenafil (9 Hz) and Tadalafil (10 Hz), demonstrating cerebral asynchrony caused by PDE inhibitors (**Fig 4A**).

In the distribution of linear power at frequencies up to 50 Hz, it was observed that the average power for the control group was 0.8766 ± 0.1009 mV^2^/ Hz × 10^−3^, which presented a statistical difference to the Sildenafil group with an average of 1.096. ± 0.1345 mV^2^/Hz × 10^−3^ (p = 0.0021). Statistical differences were also observed in the group treated with Tadalafil which averaged 1.172 ± 0.1256 mV^2^/Hz × 10^−3^ (ANOVA Tukey post-test F = 14.48) (p<0.001). There was no statistical difference between the treated groups (**Fig 4B**).

**Fig 5:**
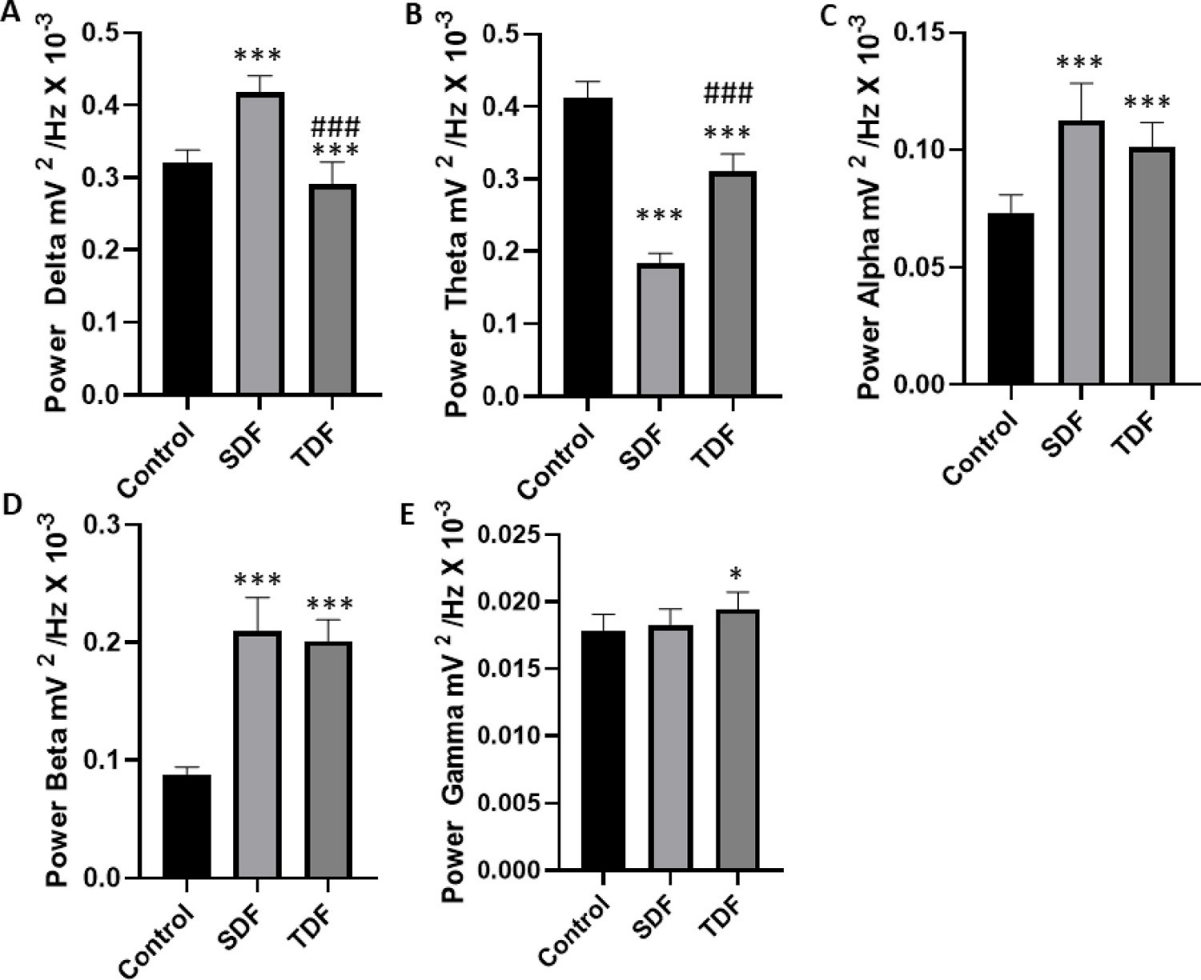
Graphs demonstration of the variation of linear power averages for brain oscillation bands. (A) Brain oscillations in the Delta band (1.0–4.0 Hz), (B) Brain oscillations in the theta band (4.0–8.0 Hz). (C) Variation in alpha oscillations (8–12 Hz), (D) Frequency oscillation in the beta band (12–28 Hz), (E) Variation in gamma Oscillations (28–40 Hz). (SDF) Sildenafil group, (TDF) Tadalafil group, (Control) Control group; (*) indicates a statistical difference between the control group, (#) indicates a statistical difference for the SDF group. (ANOVA and Tukey’s post-test) *p <0.05, ** p<0.01, *** p<0.0001 (n = 9).

The analysis of delta power averages showed greater energy intensity measured in the Sildenafil-treated group (**Fig 5A**). The analysis of delta oscillations showed that the mean of the control group was 0.3565 ± 0.04375 mV^2^/Hz × 10^−3^, which presented a statistical difference when compared to the group treated with Sildenafil with a mean of 0.4213 ± 0.02084 mV^2^/Hz × 10^−3^, revealing an increase in this parameter (p<0.001). A statistical difference was also observed for the treatment with Tadalafil, whose mean was 0.2869 ± 0.03019 mV^2^/Hz × 10^−3^ with a decrease in the power of delta oscillations for Tadalafil (p<0.001). When compared to the treated groups, there was a statistical difference indicating the dominance of delta oscillations for Sildenafil (**Fig 5A**).

The analysis of the power averages in theta showed the highest energy intensity for the control group (**Fig 5B**). The analysis of theta oscillations showed that the mean of the control group was 0.4123 ± 0.02223 mV^2^/Hz × 10^−3^, which presented a statistical difference for the Sildenafil group (0.1846 ± 0.01326 mV^2^/Hz × 10^−3^); the Tadalafil group whose mean was 0.3107 ± 0.02359 mV^2^/Hz × 10^−3^ maintained a statistical difference from the control group. When comparing the Sildenafil group with the Tadalafil group, a statistically significant difference was observed (p < 0.001) (**Fig 5B**).

From the increase in the frequency bands in alpha, synchronization between the PDE inhibitor agents occurred, with an increase in power starting at 9 Hz for Sildenafil and 10 Hz for Tadalafil (**Fig 4A**). When analyzing the alpha oscillations linearly, the control group’s mean registry was 0.07278 ± 0.008246 mV^2^/Hz × 10^−3^, which presented statistical differences to the Sildenafil group with a mean of 0.1128 ± 0.01563 mV^2^/Hz × 10^−3^ and Tadalafil with a mean of 0.1012 ± 0.01066 mV^2^/Hz × 10^−3^ (p<0.001). When compared, the Sildenafil and Tadalafil-treated groups did not show significant statistical differences between them (p = 0.0920), as shown in **Fig 5C**.

The analysis of power means in beta showed the largest difference in energy intensity for the Sildenafil and Tadalafil groups about the control. The Sildenafil and Tadalafil-treated groups did not differ in intensity (p = 0.6022), but both had intensities far above the registries from the control group, as shown in **Fig 5D**. When analyzing beta oscillations linearly, the control group’s mean was 0.08700 ± 0.007088 mV^2^/Hz × 10^−3^, which presented a statistical difference compared to the Sildenafil-treated group with a mean of 0.2095 ± 0.02854 mV^2^/Hz × 10^−3^ and treated with Tadalafil of 0.2009 ± 0.01841 mV^2^/Hz × 10^−3^ (p<0.001).

When analyzing gamma oscillations linearly the control group means was 0.01786 ± 0.001188 mV^2^/Hz × 10^−3^, it showed no statistical difference in comparison to the Sildenafil group (0.01822 ± 0.001221 mV^2^/Hz × 10^−3^) (p = 0.7801). The Tadalafil group (0.01945 ± 0.001243 mV^2^/Hz × 10^−3^) showed a statistically significant difference compared to the control group (p < 0.0182) as shown in **Fig 5E**. The treated groups showed no statistical difference (p = 0.801).

**Fig 6:**
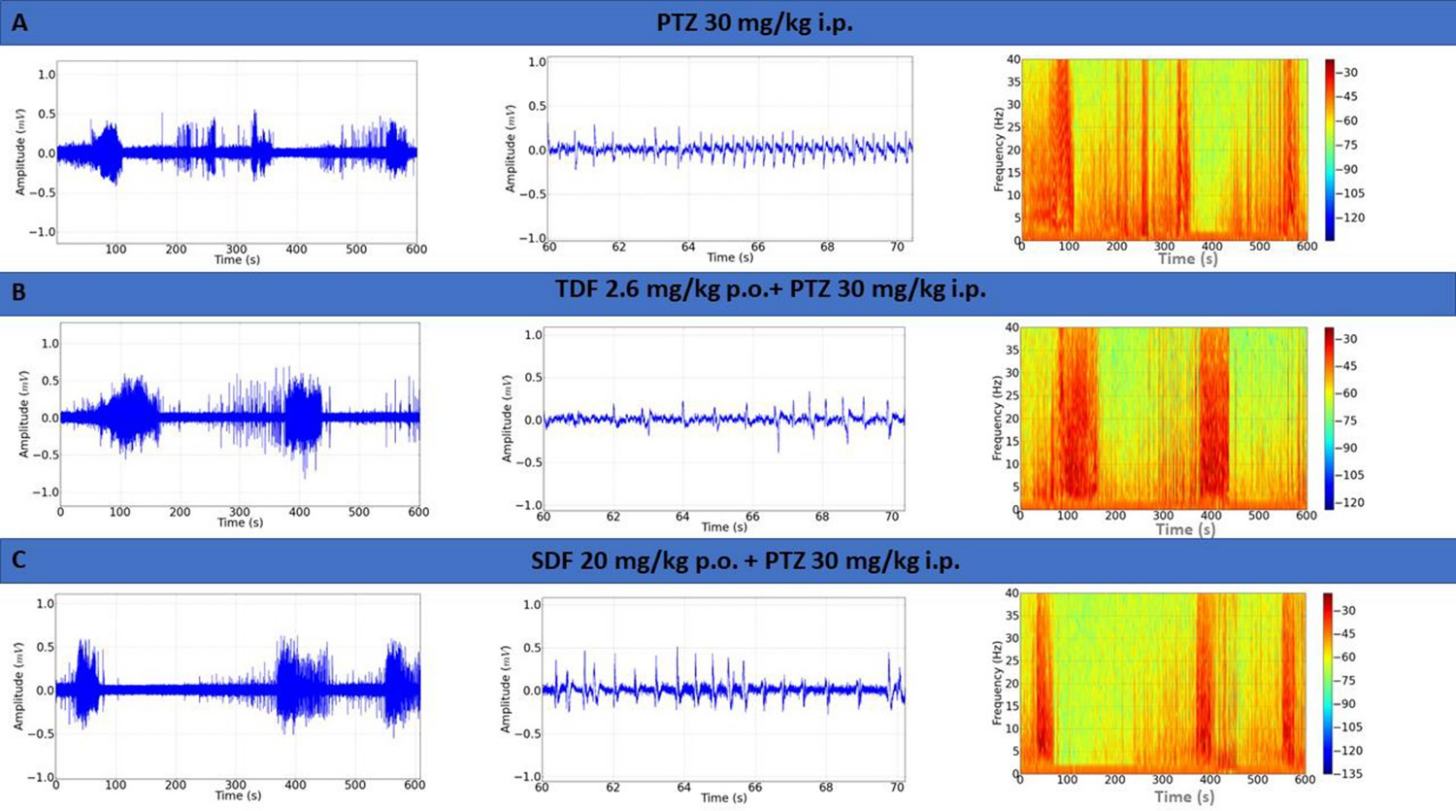
Electrocorticographic (ECoG) tracing of a Wistar rat after drug application. (A) ECoG recording after administration of Pentylenetetrazole (PTZ) (left), 10-second amplification of the tracing (60–70 s) (centre), recording spectrogram (right); (B) ECoG demonstrative recording after administration of Tadalafil (TDF) followed by application of Pentylenetetrazole (PTZ) (left), recording amplification (centre), recording spectrogram (right); (C) ECoG demonstrative recording after administration of Sildenafil (SDF) followed by application of PTZ (left), recording amplification (centre) and corresponding spectrogram (right).

ECoG tracings obtained after the application of PTZ showed irregularity and changes in amplitude with the presence of clusters of potentials; the amplification demonstrates spike waves in the recordings, and the spectrogram demonstrates the concentration of energy in the clusters of the potentials **Fig 6A**. After the use of Tadalafil, the seizure induction with PTZ maintained the characteristics of a longer duration of potential burst, the irregularities observed during amplification, and observed in the spectrogram (**Fig 6B**). After the use of Sildenafil, PTZ induction of seizures showed characteristics such as increased amplitude, with longer duration of potential bursts than the PTZ group, which can be observed with spikes in the amplification and energy concentration in the spectrogram (**Fig 6C**).

**Fig 7:**
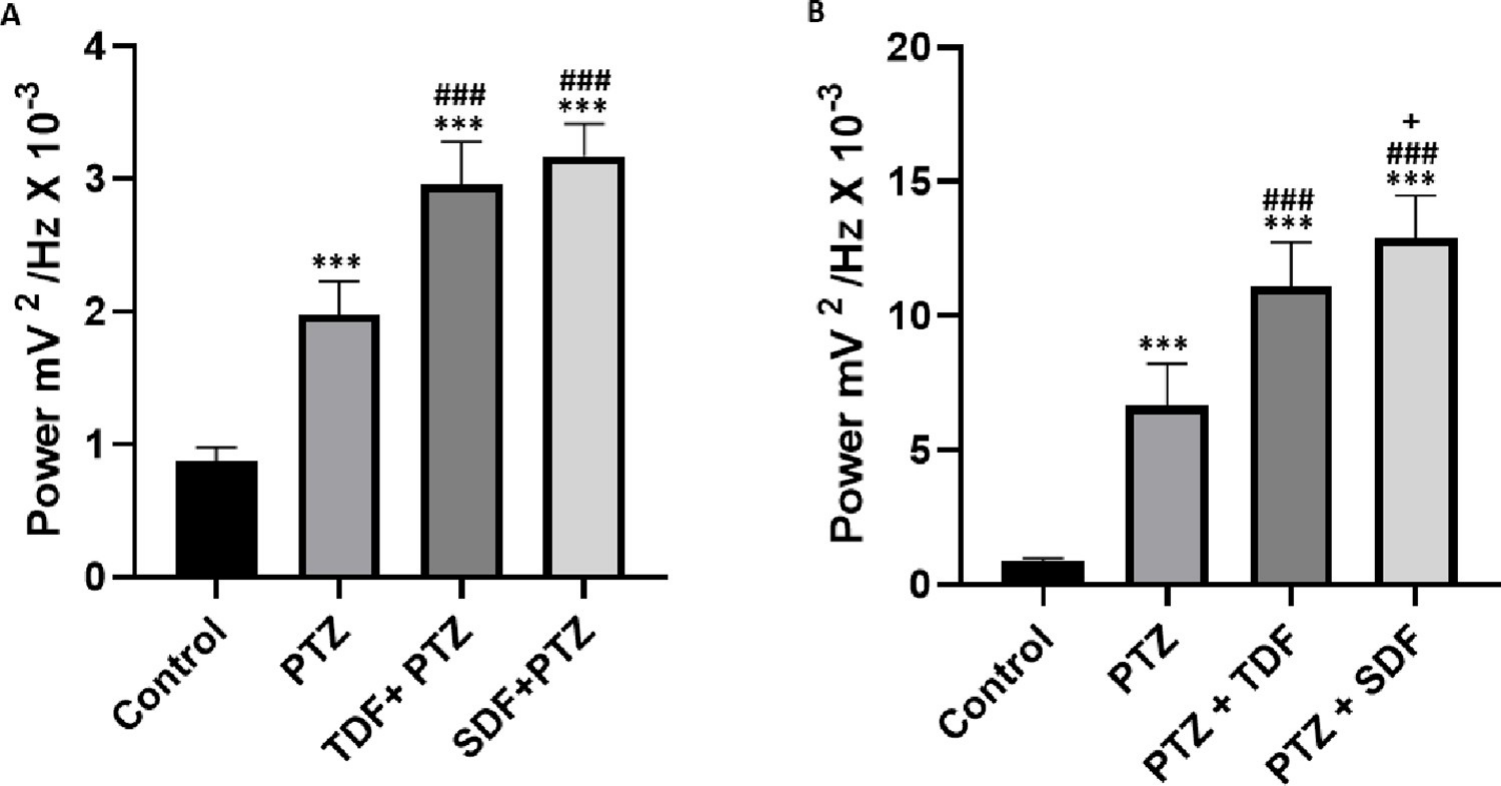
Mean linear power of recordings for groups. (A) During the Pentylenetetrazole-induced seizure; (B) During potential salvo in PTZ-induced seizure recordings. Statistical differences were obtained after ANOVA and Tukey’s test (n = 9). (*) indicates statistical difference for the control group, (#) indicates statistical difference for PTZ, and (+) indicates statistical difference for the Tadalafil +PTZ group. (Control) Control group, (PTZ) Pentylenetetrazole, (TDF) Tadalafil, (SDF) Sildenafil. The p values between the mean amplitudes are represented by asterisks *p<0.05, **p<0.01 and ***p < 0.001.

In the graph of linear power distribution in the record for the control group with a mean (0.8766 ± 0.1009 mV^2^/Hz × 10^−3^), there was a statistical difference for the PTZ group (1.974 ± 0.2527 mV^2^/Hz × 10^−3^) (p<0.001). The Tadalafil plus PTZ group presented a mean (2.957 ± 0.3201 mV^2^/Hz × 10^−3^) that presented a statistical difference for the control and PTZ groups (p<0.001). The Sildenafil and PTZ group with a mean (3.172 ± 0.2428 mV^2^/Hz × 10^−3^) showed a statistical difference for the control and PTZ groups (p<0.001) (**Fig 7A**).

Considering the volleys of potentials caused by PTZ, it can be observed that the Tadalafil PTZ group (11.10 ± 1.647 mV^2^/Hz × 10^−3^) had a significantly lower mean potency than the Sildenafil PTZ group (12.88 ± 1.600 mV^2^/Hz × 10^−3^) (p<0.05). The Tadalafil PTZ and Sildenafil PTZ groups showed a statistically significant difference when compared to the mean potency of the PTZ group (6.646 ± 1.587 mV^2^/Hz × 10^−3^) and the control group (0.8766 ± 0.1009 mV^2^/Hz × 10^−3^) (p< 0.001) (**Fig 7B**).

**Fig 8:**
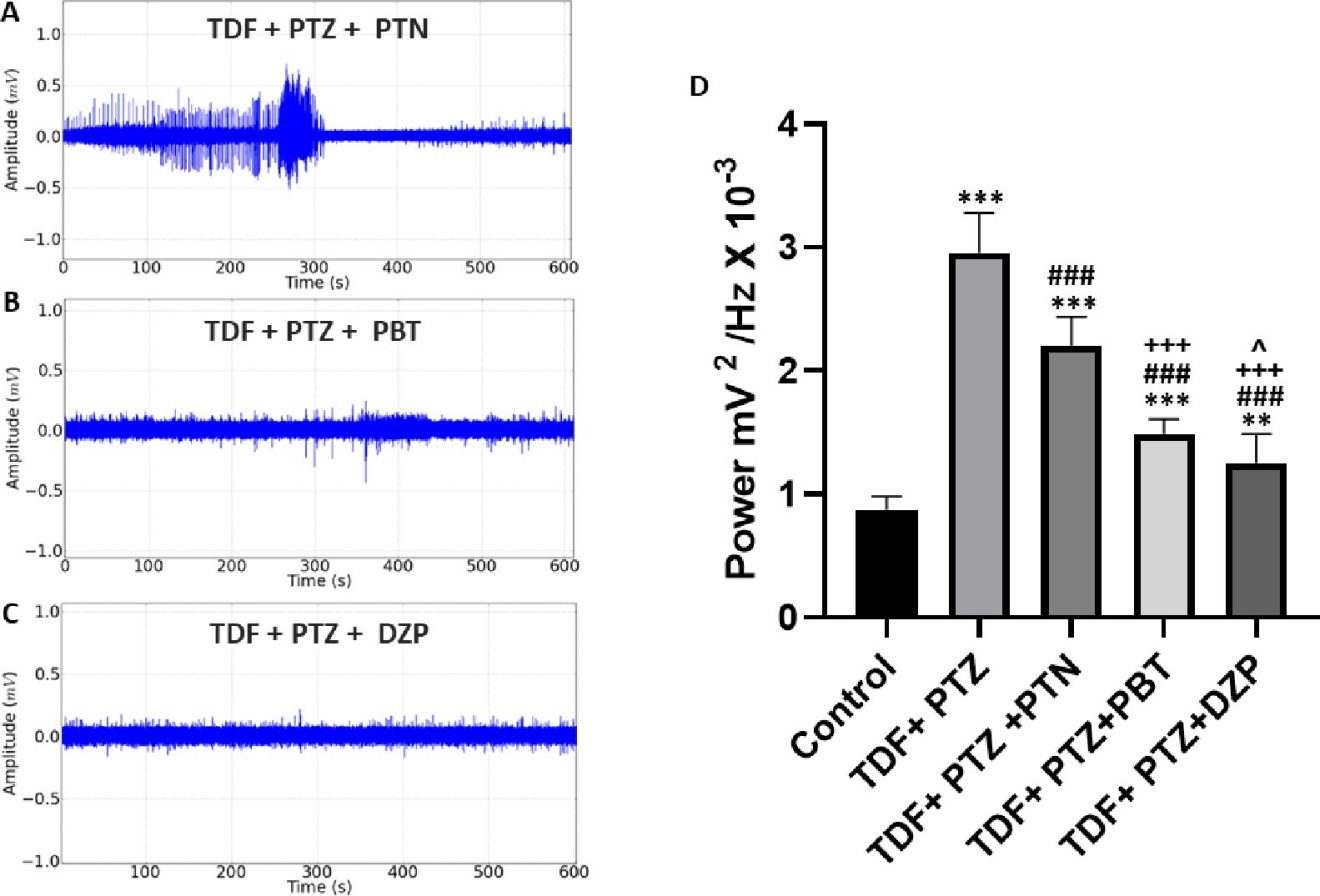
Representative electrocorticographic (ECoG) recordings were obtained after treatment with Tadalafil (TDF) followed by Pentylenetetrazole (PTZ). (A) Tracing obtained after the application of Phenytoin (PTN); (B) tracing obtained after the application of Phenobarbital (PBT); (C) Tracing obtained after application of Diazepam (DZP). (D) Graph demonstrating linear power averages of recordings after application of anticonvulsant drugs. Statistical differences were obtained after ANOVA and Tukey’s test (n = 9). (*) indicates a statistical difference for the control group, (#) indicates a statistical difference for the group that received TDF+PTZ, (+) indicates a statistical difference for the TDF+PTZ+ PTN group, and (^) indicates a statistical difference for the TDF +PTZ+ PBT group. The p values between the mean amplitudes are represented by asterisks *p<0.05, **p<0.01 and ***p < 0.001.

The control of the induced seizure after the application of Tadalafil PTZ was observed in different electrocorticographic recordings after the application of Phenytoin, Phenobarbital, and Diazepam. The tracing observed after the application of Phenytoin shows a surge of potentials at an amplitude above 0.5 mV, followed by a progressive decrease in amplitude (**Fig 8A**). The Phenytoin group (2.202 ± 0.2354 mV^2^/Hz × 10^−3^) had a lower mean potency than the Tadalafil PTZ group (2.957 ± 0.3201 mV^2^/Hz × 10^−3^) (p<0.001), also showing a difference for the group control (0.8766 ± 0.1009 mV^2^/Hz × 10^−3^) (p<0.001) (**Fig 8D**). The group that received Phenobarbital to help control seizures presented a tracing with decreased spike amplitude (**Fig 8B**). The linear power graph showed that the Phenobarbital group presented a mean potency (1.489 ± 0. 1184 mV^2^/Hz × 10^−3^), which was lower than the Tadalafil PTZ and Phenytoin group (p<0.001), and showed greater potency than the control group (p<0.001). The group that received Diazepam presented a more regular and low amplitude tracing (**Fig 8C**) presented a mean power (1.253 ± 0.2308 mV^2^/Hz × 10^−3^) presented a decrease about the Tadalafil PTZ and Phenytoin group (p<0.001), for the Phenobarbital group (p<0.05) and the control group (p<0.01) (**Fig 8D**).

**Fig 9:**
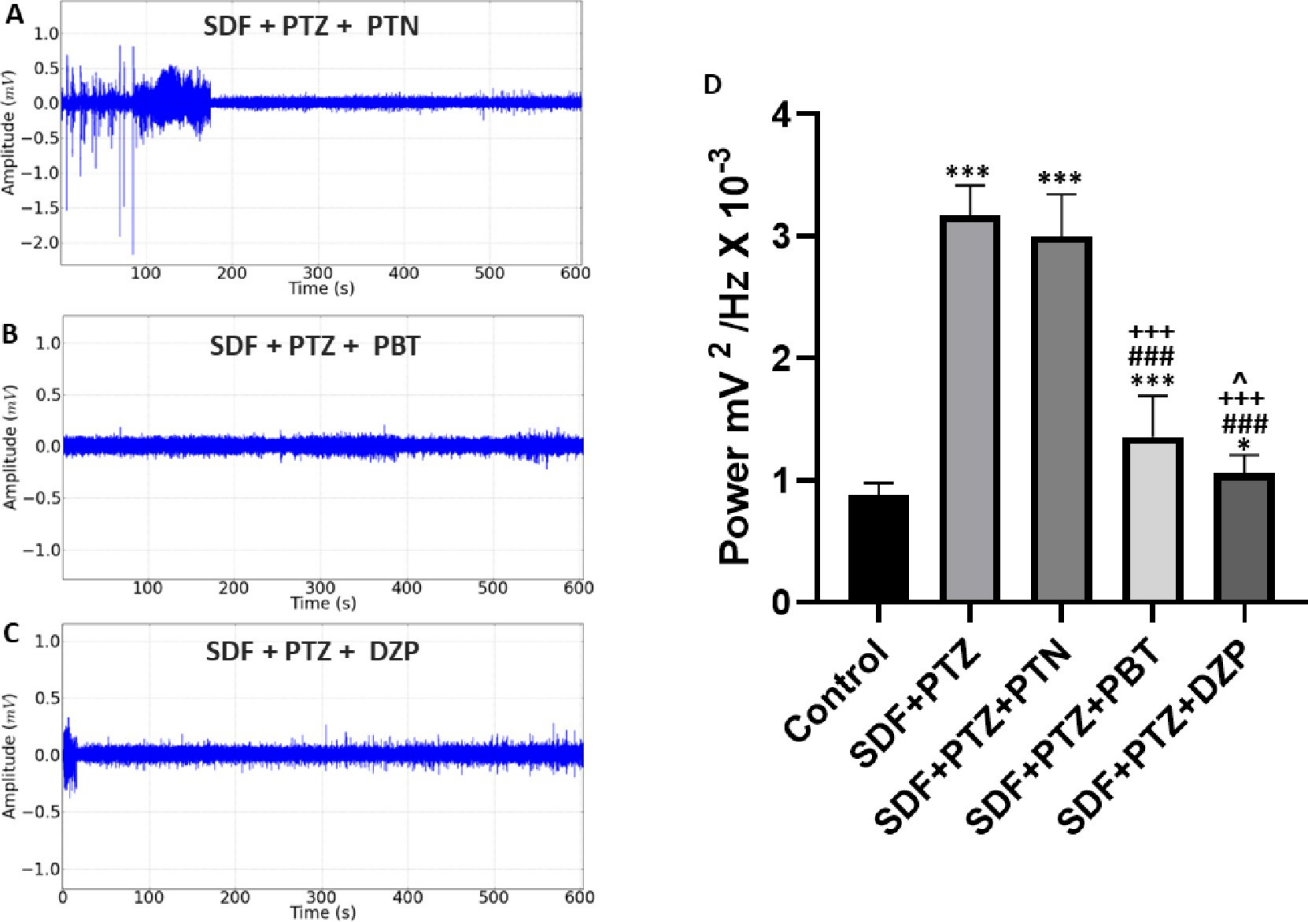
Representative electrocorticographic (ECoG) recordings were obtained after treatment with Sildenafil (SDF) followed by Pentylenetetrazole (PTZ). (A) Tracing obtained after the application of Phenytoin (PTN); (B) tracing obtained after the application of Phenobarbital (PBT); (C) Tracing obtained after the application of Diazepam (DZP); (D) Graph demonstrating linear power averages of recordings after the application of anticonvulsant drugs. Statistical differences were obtained after ANOVA and Tukey’s test (n = 9). (*) indicates a statistical difference for the control group, (#) indicates a statistical difference for the group that received TDF+PTZ, (+) indicates a statistical difference for the TDF+PTZ+ PTN group, and (^) indicates a statistical difference for the TDF group +PTZ+ PBT. The p values between the mean amplitudes are represented by asterisks *p<0.05, **p<0.01 and ***p < 0.001.

The control of the induced seizure after the application of Sildenafil and PTZ was observed in **ECoG** recordings after the application of anticonvulsants. The tracing observed after the application of Phenytoin initially presented with irregularities and high amplitude above 0.5 mV, followed by a progressive decrease in amplitude (**Fig 9A**). The Phenytoin group (3.001 ± 0.3412 mV^2^/Hz × 10^−3^) showed a mean potency with no statistical difference for the Sildenafil and PTZ group (3.172 ± 0.2428 mV^2^/Hz × 10^−3^) (p = 0.2938), showing a difference for the group control (0.8766 ± 0.1009 mV^2^/Hz × 10^−3^) (p<0.001) (**Fig 9D**). The group that received Phenobarbital to control seizures presented a tracing with a decrease in the amplitude of the spikes (**Fig 9B**), was lower than the Tadalafil, PTZ and Phenytoin group (p<0.001), and showed higher potency than the control group (p<0.001). The group that received Diazepam (**Fig 9C**) showed a mean potency (1.061 ± 0.1458 mV^2^/Hz × 10^−3^) presented a decrease in potency in relation to the Tadalafil, PTZ and Phenytoin group (p<0.001), for the Phenobarbital group (p<0.01) and the control group there was a statistical difference (p<0.05) (**Fig 9D**).

## Discussion

The present work demonstrates that PDE5 inhibitors are able to decrease the seizure threshold. This finding could be related to the modulation of this drug in the nitrergic pathway.

Currently, the main clinical use of PDE5 inhibitors involves the treatment of erectile dysfunction, whose mechanism occurs through decreasing the catalytic activity of the PDE5 enzyme in the vascular smooth muscle of the penis. This potentiates the endogenous effects of NO, synthesized by nitric oxide synthetase (NOS), which activates guanylate cyclase (GC) and induces cyclic guanosine monophosphate (cGMP) production. This mechanism causes relaxation of the smooth muscles of the penis and arterial dilation, promoting erection by distending the corpora cavernosa [20, 35, 36].

As PDE5 inhibitors can cross the blood-brain barrier [7], effects on the nitrergic pathway can be expected in brain cells. In this sense, studies demonstrate the association between the nitrergic pathway and a decrease in the seizure threshold [21, 23, 37]. NO has an excitatory role in the pathogenesis of seizures [21, 37], in which the administration of NO SYNTASE antagonists (L-NAME) may increase the seizure threshold [21], and excessive NO production under pathological conditions lowers the PTZ-induced seizure threshold [37].

The variations in power intensities in brain oscillations can be caused by the use of drugs [38, 39], like hormonal disorders and testosterone replacement therapy [40]. Studies on alpha rhythm show that increased amplitude in the alpha is related to somatization severity observed in patients after accounting for the influence of anxiety and depression [41, 42]. Other studies also add consequences like worsening performance in automotive simulators among patients with increased theta, alpha, and beta waves in the occipital region [43]. Our analysis of ECoG records in rats demonstrated an increase in power in the frequency bands in alpha after the application of Sildenafil and Tadalafil. It also demonstrated the synchronization of the elevation of the powers in the brain oscillations in alpha, beta, and gamma after the application of PDE5 inhibitors.

The presence of PDE5 has been found in the rodent cerebral cortex and hippocampus regions, where administration of selective PDE inhibitors would cause accumulation of cGMP [44]. However, it is also important to highlight the possibilities of adverse effects [45]. Furthermore, decreased epileptic threshold (proconvulsant action) has been reported following the use of PDE inhibitors [15–19, 46]. According to Hamoy et al. [27] and Silva de Melo et al. [28], an increase in the frequency band in beta in the motor cortex of rats is related to the onset of seizures. This data corroborates with our results, which demonstrate that the use of Sildenafil and Tadalafil increased the power of the frequency band in beta.

The beta range is modulated by cognitive tasks that require a relationship between sensory and motor states [47, 48]. Brain stimulation by electrotherapy shows a significant increase in beta waves as well as increased alertness, focus and concentration [49]. There are disagreements between authors as scientific texts are correlating the increase of this particular frequency range with anxiety, restlessness, and irritation in sleep disorders [50, 51] or associations of this band along with theta forces in depressive conditions and chemical dependence [52]. The use of Sildenafil and Tadalafil increased brain oscillations in beta, which may be related to one or more of the various neurological disorders mentioned.

Other findings of interest include the increase in gamma wave amplitudes observed in rodents undergoing Tadalafil experiments, which was related to the increased presence of catecholaminergic neurotransmitters in the cortex [53]. It is also observed in patients who use Modafinil for the treatment of schizophrenia [54] and used for the improvement of sensory perception activities, attention and maintenance processing of memory information, and state of consciousness [47, 55].

There are also increases in delta, alpha, and beta potency in the use of heroin and morphine that exhibit an EEG profile similar to the results obtained with Sildenafil, as this drug has caused a similar change to the three waves mentioned above [56]. Work in progress on modulating micromolecule-based synaptic plasticity such as NYX-2925—modulating NMDA receptors—for the treatment of pain in diseases such as fibromyalgia and diabetic peripheral neuropathy also seeks to increase alpha waves [57]. Our work demonstrated the synchronization of increased powers in the alpha and beta bands after the use of Sildenafil and Tadalafil.

According to Sroykham & Wongsawat [58], in the analysis of the EEG of patients at rest, the proportion of delta and beta oscillations can estimate the hormonal levels of testosterone and cortisol, being biomarkers for physiological and psychological disorders.

The increase in beta potency is related to the aggressive behaviour observed in the animal, due to activity observed in the thalamic and cortical regions [40, 59, 60]. According to our data, the increase in the potency of the beta oscillations may be related to the decrease in the seizure threshold since, after the application of PTZ, there was an increase in the recorded potency for the groups that received Tadalafil and Sildenafil in advance. Milman & Arnold [61] showed evidence of an association between Sildenafil and several adverse effects on the central nervous system, including seizures.

Fast-wave EEG markers have been revealed, with the observation of increased alpha and beta activities in attention deficit hyperactivity disorder [62]. In a similar population, an increase in beta potency in the parietal and occipital lobe regions was identified after the use of methylphenidate or atomoxetine [63]. Animals receiving Sildenafil and Tadalafil showed increased alpha and beta oscillations.

Beta desynchronization is an essential process in the generation of movement [64]. Alpha is associated with the recruitment of a functional assembly required to generate the motor output [65, 66]. Therefore, when relating alpha and beta, it is possible to infer that the proportion of beta increases synchronized with alpha, observed in the spectral power distribution. Therefore, both Sildenafil and Tadalafil can cause a delay in the execution of planned movements.

According to de Carvalho et al. [20], Sildenafil lowers the epileptic threshold which could be related to increased cholinergic and nitrergic activity in the brain. Nieoczym et al. [15] noted that Sildenafil did not significantly influence the focal seizure threshold in the rat model with amygdala epilepsy, nor did it influence seizure severity and had weak anticonvulsant action. Nieoczym et al. [14], observed that there was no influence of Sildenafil on the latency of clonic seizures induced by cocaine administration, and it did not influence the incidence of mortality.

However, in the literature, there are reports of alterations in brain homeostasis related to the use of PDE5 inhibitors [24, 35, 67–69]. Our data demonstrates that both Sildenafil and Tadalafil increased the recording potency after PTZ administration. However, the recordings of the PTZ Sildenafil group showed higher energy intensity bursts when compared to the PTZ and Tadalafil groups.

Gholipour et al. [13] suggests that PDE5 inhibitors may facilitate GABA (A)-mediated inhibition and may potentiate the action of Benzodiazepine against PTZ-induced seizures. Assessing the possible decrease in seizures, anticonvulsants in the Tadalafil-PTZ and Sildenafil-PTZ groups showed Phenytoin did not adequately control the model seizures. For the Sildenafil+PTZ group, Phenytoin had no effect. Phenobarbital and Diazepam were more efficient in controlling seizures.

## Conclusion

PDE5 inhibitors altered the electrocorticographic recordings in the motor cortex of the rats, causing an increase in potency in the alpha and beta frequency bands synchronously. After the induction of seizures with PTZ, there was an increase in energy during the more persistent potential for the Sildenafil group. Phenytoin did not adequately control the model seizures and showed a better response to Phenobarbital and Diazepam. These findings indicate that the use of PDE5 inhibitors may precipitate changes in the neurological system with impairment of the nitrergic pathway.
